# Ameliorative Sexual Behavior and Phosphodiesterase-5 Inhibitory Effects of *Spondias mangifera* Fruit Extract in Rodents: *In Silico*, *In Vitro*, and *In Vivo* Study

**DOI:** 10.3390/jcm11133732

**Published:** 2022-06-28

**Authors:** Mohammad Khalid, Mohammed H. Alqarni, Shadma Wahab, Sivakumar Annadurai, Mubarak A. Alamri, Ahmed I. Foudah, Tariq M. Aljarba, Juber Akhtar, Badruddeen, Sarfaraz Ahmad

**Affiliations:** 1 Department of Pharmacognosy, College of Pharmacy, Prince Sattam Bin Abdulaziz University, Al-Kharj 11942, Saudi Arabia; m.alqarni@psau.edu.sa (M.H.A.); a.foudah@psau.edu.sa (A.I.F.); t.aljarba@psau.edu.sa (T.M.A.); 2 Department of Pharmacognosy, College of Pharmacy, King Khalid University, Abha 61421, Saudi Arabia; shad.nnp@gmail.com (S.W.); sannadurai@kku.edu.sa (S.A.); 3 Department of Pharmaceutical Chemistry, College of Pharmacy, Prince Sattam Bin Abdulaziz University, Al-Kharj 11942, Saudi Arabia; mubarak@psau.edu.sa; 4 Department of Pharmaceutics, Faculty of Pharmacy, Integral University, Dasauli, Kursi Road, Lucknow 226026, India; juberakhtar@gmail.com; 5 Department of Pharmacology, Faculty of Pharmacy, Integral University, Dasauli, Kursi Road, Lucknow 226026, India; badarmiracle@gmail.com; 6 Department of Clinical Pharmacy, College of Pharmacy, Jazan University, Jazan 45142, Saudi Arabia; sarfaraz3030@gmail.com

**Keywords:** *S. mangifera*, aphrodisiac, ejaculation, impotency, mount frequency, phosphodiesterase-5, *in-silico*

## Abstract

The ethanolic extracts of *Spondias mangifera* fruit (SMFE) were evaluated for aphrodisiac activity. The in-vitro phosphodiesterase-5 (PDE-5) inhibition was assessed based on in-silico molecular docking and simulation studies. In addition, the in-vivo sexual behavior was analyzed in the form of mount (MF, ML), intromission (IF, IL), and ejaculation (EF, EL) frequencies and latencies to validate the in-vitro results. Some biochemical parameters, including PDE-5, nitric oxide, and testosterone, were also observed. The above extract constituted β-amyrin, β-sitosterol, and oleanolic acid and showed tremendous binding with phosphodiesterase-5 and sildenafil. Both the sildenafil and ethanolic extracts (200 and 400 mg/kg/d bodyweight) significantly (*p* < 0.1, *p* < 0.05) increased MF, IF, and EF, respectively. In contrast, ML and IL significantly (*p* < 0.1) decreased, and EL significantly (*p* < 0.1) increased compared with a normal group of animals. The ethanolic extracts (200 and 400 mg/kg/d bodyweight) and sildenafil further significantly (*p* < 0.05, *p* < 0.1) diminished PDE-5 activity significantly (*p* < 0.05, *p* < 0.1) and enhanced nitric oxide and testosterone levels, as compared with normal rodents. Therefore, the *S. mangifera* ethanolic extract might be a valuable alternate aphrodisiac for erectile dysfunction.

## 1. Introduction

Sexual dysfunction is now considered a persistent disorder, particularly among those aged 40 to 70, owing to various physical, psychological, aging, and lifestyle causes [1,2]. People with infertility are more likely to have early ejaculation, reduced sexual desire, and erectile dysfunction [3]. The number of men with erectile dysfunction is expected to rise to nearly 300 million by 2025 [4]. Sexual performance and motivations are regulated by the hypothalamic-pituitary-gonadal axis [5]. An aphrodisiac affects sexual function, arousal, or both by modulating blood flow to the reproductive organs through the central nervous system or the peripheral nervous system [6]. The most commonly used allopathic aphrodisiac drugs are sildenafil citrate, phosphodiesterase-5 inhibitors, α2-adrenergic receptor antagonists, and dopamine receptor agonists [7], which are used to achieve sexual desires. Still, they can be associated with specific side effects such as painful erection, visual disturbance, hypotension, diarrhea, flushing, and dyspepsia, respectively, necessitating the search for alternate medications or sources [8].

Medicinal herbs have been used for ages to produce safe and effective treatments in contemporary and traditional healthcare systems [9,10,11]. The herbal medicines are helpful for the management of sexual dysfunction. There are a lot of herbal remedies for treating erectile dysfunction in traditional medicine [12]. It has been shown that traditional remedies and formulations that include extracts of *Ginkgo biloba* L. and *Vitis vinifera* L. are also known to be inhibitors of the PDE5 enzyme [3,13]. In addition, the effectiveness of several herbs such as *Polygonatum verticillatum* [14], *Allium tuberosum* [15], *Carpolobia alba* [16], *Piper guineense* and *Zanthoxylum leprieurii* [17], and *Salvia haematodes* [18] have been experimentally shown to improve sexual activity in animal models. Therefore, computational molecular modeling has become essential for developing drugs from natural products. Docking refers to determining the geometry of the binding that occurs between two interacting molecules that have previously established structures. Docking predicts the optimal orientation of two molecules linked together to create a stable complex [19].

*Spondias mangifera* belongs to the family Anacardiaceae, also known as Amrata, which are small woody and aromatic plants. The states Punjab, West Bengal, Maharashtra, Assam, and Orissa in India are the primary locations for the cultivation of this plant to produce edible fruits [20]. The vitamin content of the mature fruit juice of this plant makes it a potential nutraceutical agent [21]. Unani and Ayurveda, two Indian medical systems, have recorded the traditional use of almost all tree parts for home treatments against many human ailments [22]. People in the north area of India utilized it as a rheumatism cure in ancient times [23]. Fruit powder of the plant is used to treat dysentery [22]. Additionally, the plant’s fruits and barks are used to treat diabetes [24]. The leaves are used in food seasoning due to their fragrant qualities and astringent and acidic properties, whereas the juice is employed to cure earaches [25,26]. In mice, methanolic extracts of stem heartwood have shown hepatoprotective effects against carbon tetrachloride-induced liver impairment [27]. *S. mangifera* fruits and aerial parts contain beta-sitosterol, oleanolic acid, lignoceric acid, beta amyrin dausterol cycloartanone-24-methylene, and daucosterol. It also constitutes small amounts of other bioactives such as galloylgeranin, alanine, niacin, and riboflavin [28].

The evidence in the literature of the use of nutraceuticals for erectile dysfunction is encouraging but preliminary [29]. *S. mangifera* fruit extract has antioxidant potential and exhibits significant activity against the alpha-glucosidase enzyme [30]. This provides substantial evidence for using *S. mangifera* fruits to treat diabetes mellitus, as in diabetic individuals, hyperglycemia leads to sperm abnormalities and even erectile dysfunction [31,32]. In this way, it might be used to improve fertility. *S. mangifera* has received very little scientific attention despite its widespread usage in traditional medicine in northern India. The above-mentioned findings led us to hypothesize that *S. mangifera* fruit may similarly ameliorate sexual behavior. Therefore, this work intended to evaluate ameliorative sexual behavior and the phosphodiesterase-5 inhibitory effects of *S. mangifera* fruit extract in rodents. With concerns for assessing the plant’s aphrodisiac effect, first, the active constituents of *S. mangifera* fruit were evaluated in molecular docking research to assess their binding affinity. Then, further docking results were validated through *in vitro* and *in vivo* experiments.

## 2. Materials and Methods

### 2.1. Plant Authentication

Fruits were collected from the local market of Aminabad, Lucknow, India. The collected fruits were identified by Dr Muhammad Arif, Department of Pharmacognosy, Faculty of Pharmacy, Integral University, Dasauli, Kursi Road, Lucknow, India. For future reference, a voucher specimen was submitted (IU/PHAR/HRB/20/16).

### 2.2. Chemicals

All of the chemicals used in this study were of analytical grade and purchased from Sigma Aldrich (St. Louis, MO, USA).

### 2.3. Molecular Docking

This study used auto dock, vin 4.2 software (The Apache Software Foundation, 1000 N West Street, Suite 1200 Wilmington, DE 19801, USA) to conduct docking analyses. The PDB file of the X-ray crystal structure of PDE-5 in complex with SLD (PDB ID: 2H42, 2.30 Å) and PDB ID: 6CM4, resolution 2.87 Å were downloaded from https://www.rcsb.org/structure, (accessed on 15 April 2022). The PDB was utilized for molecular docking studies [33]. The heterogenous and water molecules were removed to perform the docking study. Affinity grid 60 Å × 60 Å × 60 Å were produced using the active binding site residues in the protein structure. The outline employing “Auto grid” aimed to target pocket binding. The *in silico* docking studies predicted aphrodisiac activity due to bioactive constituents such as alpha amyrin, oleanolic acid, lignoceric acid, and beta-sitosterol in *S. mangifera* fruit extract. The structure of bioactive phytoconstituents (β-amyrin, β-sitosterol, and oleanolic acid) were prepared from chem draw version 12.0 (Version 12.0.2, licensed: Mulder 2010 Cambridge soft, Cambridge, MA, USA) and converted into an SDF mole file. The binding affinity (Kd) of phytoconstituents towards proteins was estimated utilizing the subsequent relation [34].

### 2.4. Molecular Dynamics (MD) Simulation

MD simulations for compounds were run by the package systems of molecular Dynamic (MD) Simulation GROMACS 2018.1, as previously reported [30]. Docked β-amyrin and co-crystallized sildenafil in complex with Phosphodiesterase-5 (PDE5) (PDB ID: 2H42) were simulated by an OPLS-AA all-atom force field using the TIP3P water model. Online Swiss PARAM web services were used for generating the topological parameters for ligands [35]. A cubic box with at least 1 nm spacing from the protein-ligand combination was utilized to solvate and neutralize the complexes. An amount of 0.15 M of counter ions were employed, which were constituted of (Na^+^/Cl^−^). To reduce the energy consumption of the complexes, a steepest descent algorithm technique was adopted. The tolerance was set at 1000 kJ/mol/nm with a step size of 0.01 nm as the maximum. The Linear Constraint Solver technique (LINCS) was employed to set bond length constraints. Electrostatic calculations were carried out using the particle mesh Ewald (PME) method. NVT and NPT (isobaric–isomerical ensemble) canonical ensembles were used for 100 ps equilibration, and the 100 ns production run followed. Root-mean square fluctuations (RMSF), root-mean-square deviation (RMSD), a radius of gyration (Rg), and potential energy were analyzed using a GROMACS 2018.1 package toolkit.

### 2.5. Preparation of Extracts

Initially, the collected fruits were cut into four small pieces and scattered open-air at room temperature for drying. The dried fruits were further chopped with the chopper and dried in an oven for 2–3 days at 40–45 °C until a constant weight was obtained. The dried materials so obtained were made into coarse powder by the grinder. The coarse powder was defatted with petroleum ether (40–60 °C) and macerated with 80% ethanol for 72 h. Then, the extract (SMFE) was filtered using Whatman filter paper and concentrated up to dryness at 40 °C with the help of a rotary evaporator. It was kept in the refrigerator for further use [36].

### 2.6. Animals

After getting the approval from the Institutional Animal Ethics Committee (IAEC), (Approval No.: IU/CPCSEA/08/ac/1213), the fresh albino healthy mice (30–40 ± 5 g) were brought from the animal house of the National Laboratory Animal Centre (NLAC), Central Drug Research Institute, Lucknow, India, housed separately in polypropylene cages at room temperature (21–27 °C)and relative humidity with the light-dark cycle of 12/12 h for one week. These were acclimatized and fed with pellets and water ad-libitum.

#### 2.6.1. Experimental Protocol

The mice were randomly divided into five groups. Group I served as normal saline, group II received sildenafil citrate (standard) 5 mg/kg suspended in 0.5% carboxyl methylcellulose (CMC), group III, IV, and V animals received (at doses of 100, 200, and 400 mg/kg) *S. mangifera* fruits extract, respectively, for 28 days. Sexual behavioral parameters were recorded after the experimental period.

#### 2.6.2. Preparation of Male Animals

Before the behavioral study, the virgin male animals were acclimatized for 3–4 weeks. The single male mice were paired with the single female mice. After 15 min of acclimatization, the animals were observed for 30 min. The study was conducted under dim red light in a silent room [37].

#### 2.6.3. Preparation of Female Animals

The female mice were treated with progesterone (1 mg/kg subcutaneously) so that the oestrus phase started after 4 h of the above treatment [7,38]. The female mice were kept in the glass case and observed. After 1 h of acclimation, the male mice were introduced to a female cage for 30 min. On the 1st, 7th, 14th, 21st, and 28th days of treatment, sexual activities were observed [8,39].

### 2.7. Study Protocol for Male Sexual Behaviors

The test was carried out by acclimating the male mice in a plastic cage (60 × 40 × 40 cm) for 1 h. Female mice were placed into the male mice cage after 10 min of estrus. The findings were as follows: (a) Mount latency is the time that elapses between the insertion of the female and the initial mounting by the male. (b) The number of mounts that remain unintroduced from the moment a female is introduced until the moment of ejaculation is called the “mount frequency”. (c) The interval between the moment of female introduction and the first intromission by the male is known as intromission latency (also known as intromission lag). (d) Intromission frequency measures how many intromissions occur between the moment a female is introduced and the time at which ejaculation occurs. (e) The period between the initial intromission to ejaculation is called ejaculation latency, which follows a period of immobility, time-consumption, deep pelvic pushing, and an unhurried dismount. (f) The number of ejaculations in a session is called ejaculation frequency [37].

### 2.8. Determination of Phosphodiesterase-5 (PDE-5)

A PDE-Glophosphodiesterase Assay kit was used for the determination of phosphodiesterase. The penis was isolated and mixed with lysate RIPA buffer solution centrifuged at 4 °C for 15 min. The clear supernatant was extracted according to the kit’s methodology to evaluate PDE-5 activity. The phosphodiesterase reaction was completed by incubating the penis with cyclic guanosine monophosphate (cGMP) substrate. A PDE detection solution comprising adenosine triphosphate (ATP) and protein kinase A was treated with the PDE-Glotermination buffer (PKA). The luciferase-based Kinase-Glo reagent was used to measure the quantity of ATP used by this reaction, which was directly associated with the level of cGMP. The optical density of the sample was evaluated using a SpectraMax L microplate luminometer (MDS AT (US) Inc., Stuart, FL, USA) after a 10-min incubation time at room temperature and was expressed as a percentage of the control and relative light units (RLUs) [40,41].

### 2.9. Testosterone Serum Preparation

Mice were anesthetized with ketamine hydrochloride, and approximately 3 to 5 mL of blood was collected without anticoagulant-containing tubes with the help of a sterile syringe. The tubes were retained for coagulation and monitoring for 5 to 10 min. Then, the tubes were centrifuged at 3000 rpm for 10 to 15 min. Finally, the liquid from the supernatant was pumped into clean tubes to determine testosterone levels [42,43].

### 2.10. Testosterone Analysis

The competitive calorimetric method was performed for the testosterone assay in supernatant liquid according to the given procedure by the manufacturer (Diametra testosterone ELISA kit). At 420 nm, absorbance was measured against a reference wavelength of 620–630 nm.

### 2.11. Measurement of Nitric Oxide Level

During the experiment, the penile tissues were collected and homogenized with a liquid comprising 400 mL of % vanadium chloride in % hydrochloric acid, 200 mL of % *N*-(l-naphthyl) ethylenediamine dihydrochloride, and 200 mL of % sulphanilamide (in % hydrochloric acid, respectively). It was incubated for an hour at the same temperature (37 °C). The conversion of nitrate to nitrite by CCl_3_ is the basis for this approach. The nitrite level, which corresponds to an estimated nitrate level, was measured spectrophotometrically at 540 nm after incubation [42].

## 3. Results

### 3.1. Molecular Docking Analysis

**Figure 1:**
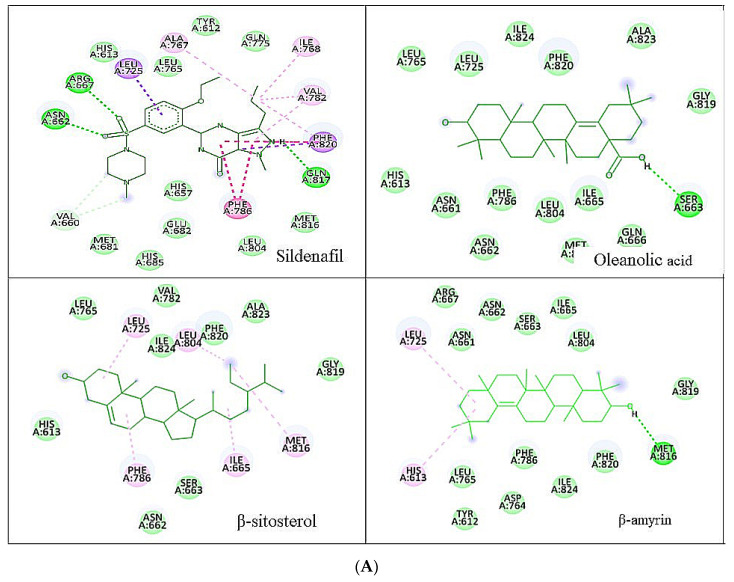

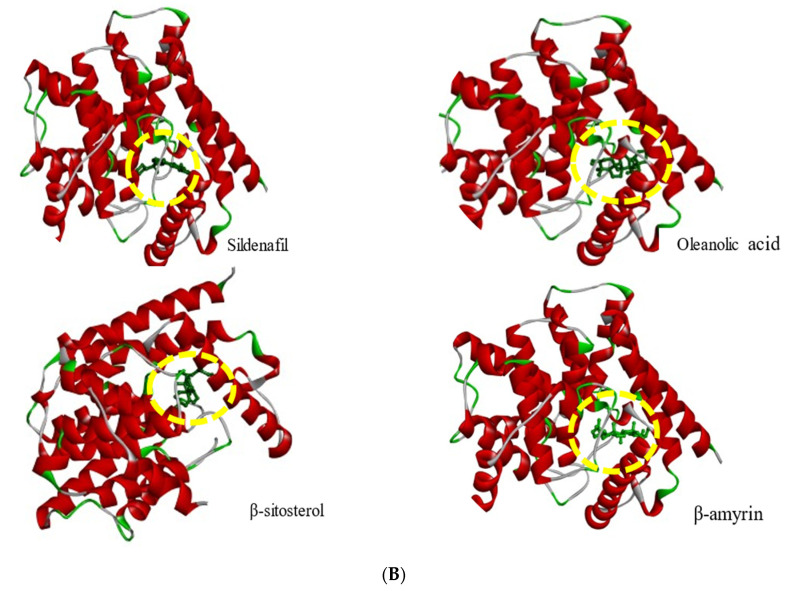
(**A**) Two-dimensional representation of the docked conformation of PDE-5 with ligands obtained after AutoDock vin docking. Green dashed lines represent conventional hydrogen bonds with the interacting amino acid residues. Pink lines indicate interactions between hydrophobic (alkyl–alkyl, π–alkyl or π–π). (**B**) Three-dimensional representation of the docked conformation of the PDE-5 representation of docked conformation with ligands obtained after docking.

**Figure 2:**
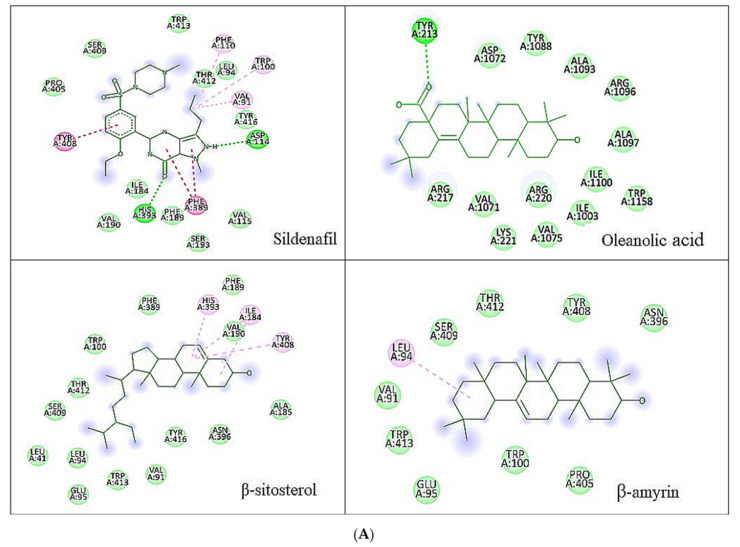

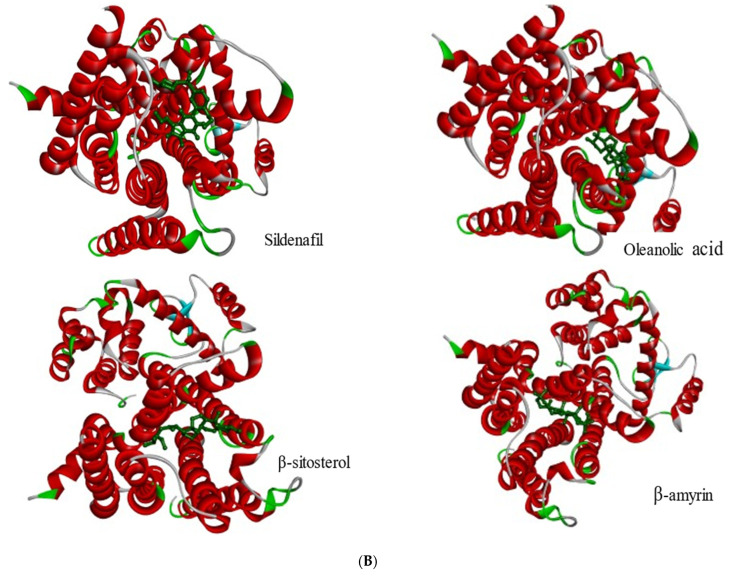
(**A**) Two-dimensional representation of the docked conformation of dopamine R_2_ with ligands obtained after docking. Green dashed lines represent conventional hydrogen bonds with the interacting amino acid residues. Pink lines indicate interactions between hydrophobic (alkyl–alkyl, π–alkyl, or π–π). (**B**) Three-dimensional representation of the docked conformation of dopamine R_2_ docked conformation of ligands obtained after Glide XP docking.

β-amyrin, β-sitosterol, and oleanolic acid were docked into the active site of phosphodiesterase-5 to provide insight into the mechanism of action of the key ingredients of *S. mangifera* using (PDB ID: 2H42, resolution 2.30 Å) and dopamine R D_2_ (PDB ID: 6CM4 resolution 2.87 Å) by AutoDock vin 4.2. (The Apache Software Foundation, 1000 N West Street, Suite 1200 Wilmington, DE 19801, USA) When compared to the dopamine receptor D2, the key ingredients such as β-amyrin, β-sitosterol, and oleanolic acid demonstrated a superior binding affinity for phosphodiesterase-5. Our docking methodology was validated when the co-crystallized ligand sildenafil exhibited a comparable binding posture to the crystal structure. β-amyrin, β-sitosterol, oleanolic acid, and sildenafil shared high similarities in their interactions with phosphodiesterase-5 and dopamine RD2 in their corresponding bound states. The AutoDock score and binding residues are shown in Table 1 and Table 2. The 3D and 2D illustrations of the binding view of β-amyrin, β-sitosterol, oleanolic acid, and sildenafil are presented in Figure 1A,B and Figure 2A,B, respectively. Structural analysis of the best complex was conducted using the Discovery Studio visualizer tool.

### 3.2. Binding Stability Analysis Using MD Simulation

**Figure 3:**
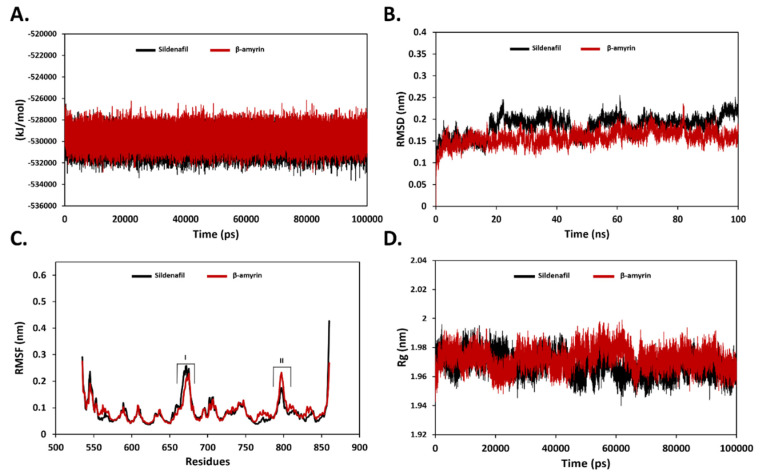
MD simulation plots for checking the overall stability of the systems during 100 ns. (**A**) Potential energy profile of PDE5 in the presence of sildenafil and β-amyrin. (**B**) RMSD profile of PDE5 in the presence of sildenafil and β-amyrin. (**C**) RMSF of simulated complexes of PDE5 with sildenafil and β-amyrin. (**D**) Radius of gyration of PDE5 with bound sildenafil and β-amyrin. The figure was drawn using Microsoft Excel 2016.

Molecular dynamic simulations of 100 ns for compounds complexed with PDE5 were performed to investigate the effect of explicit water solvent on the stability of reference compounds: (co-crystallized sildenafil) and β-amyrin in the catalytic pocket of PDE5. The simulations were evaluated to assess the binding affinity of the top pose of docked β-amyrin towards PDE5. The conformational stability of PDE5 in complex with compounds was investigated by calculating the potential energy of the systems as well as the root mean square fluctuations (RMSF) and root mean square deviation (RMSD) of the PDE5 backbone atoms and side chains of residues, respectively (Figure 3). The minimum potential energy of both simulated systems was low at ~−534,000 kJ/mol, showing that both complexes were energetically stable during the production MD run (Figure 3A). The RMSD profiles of both systems showed that, in the first 2 ns, the protein backbone rose to equilibrate and then remained relatively stable until the 100th ns of production MD with a maximum RMSD value of ~0.25 nm. Compared to β-amyrin, sildenafil illustrated a more fluctuated pattern and higher RMSD values (between 0.15 to 0.25 nm) after the first 20 ns. This longest period of equilibration for both systems can confirm the prolonged stability of the PDE5 in the presence of the compounds (Figure 3B).

Moreover, the RMS fluctuation of PDE5 was also calculated, showing three flexible regions (I, II and III) (Figure 3C). The first flexible region (I) is the short α-helix (672–677) located in the H-loop, which is juxtaposed to the catalytic pocket of the PDE5, with a maximum RMSD deviation of ~0.2 nm [44]. The second flexible region (II) is also located in a loop region (795–800) connecting the α-14 and α-15 helices, with a maximum RMSD ranging from 0.2 to 0.25 nm. Furthermore, the compactness of PDE5 in complex with compounds was also assessed by calculating the radius of gyration (Rg) (Figure 3D). The overall Rg values (1.96 nm to 2.0 nm) of PDE-5 suggest the compactness of PDE-5 during simulations due to the stable behavior of protein secondary structures.

### 3.3. Effect of S. mangifera Ethanolic Extract on the Mount, Intermission, and Ejaculatory Frequencies

**Figure 4:**
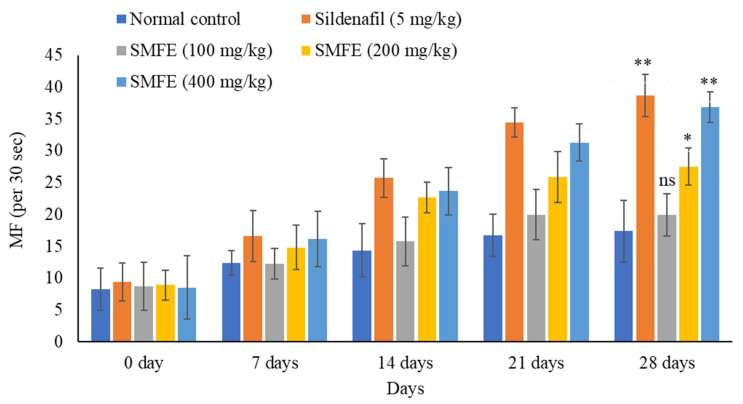
Effect of *S. mangifera* fruit extract on mount frequency (*n* = 6). The values are means ± SEM; * *p* < 0.05, ** *p* < 0.1; ^ns^ *p* > 0.05 (followed by Student Dunnett test).

**Figure 5:**
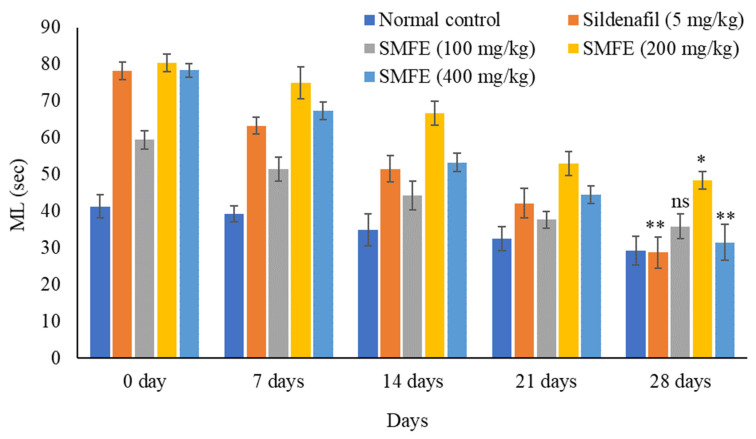
Effect of *S. mangifera* fruit extract on mount latency (*n* = 6). The values are means ± SEM; * *p* < 0.05, ** *p* < 0.1; ^ns^ *p* > 0.05 (followed by Student Dunnett test).

**Figure 6:**
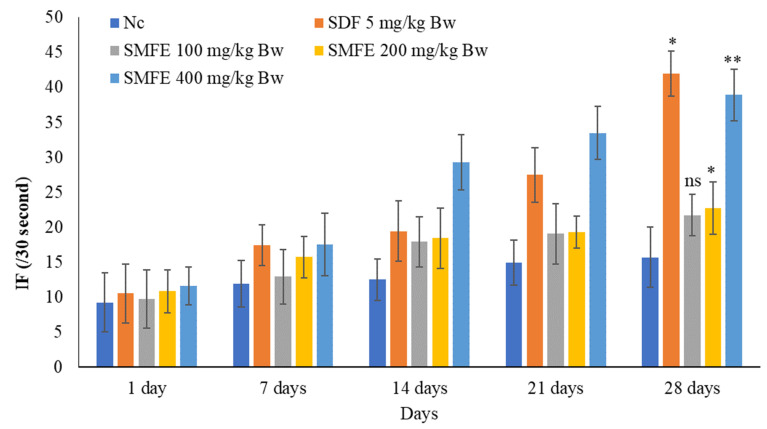
Effect of *S. mangifera* fruit extract on intromission frequency (*n* = 6). The values are means ± SEM; * *p* < 0.05, ** *p* < 0.1; ^ns^ *p* > 0.05 (followed by Student Dunnett test).

There was a significant (*p* < 0.1) increase in MF, IF, and EF after sildenafil citrate treatment (5 mg/kg/d bodyweight) in comparison to normal control animals. The ethanolic extract of *S. mangifera* fruits was given to the mice at 200 and 400 mg/kg/day. On day 28, the MF, IF, and EF rose considerably (*p* < 0.1, *p* < 0.05) at 400 mg/kg/d, but the effects were less significant (*p* < 0.05) at 200 mg/kg/d. However, 100 mg/kg/d bodyweight did not show significant (*p* > 0.05) effects on the above parameters when compared with normal-treated animals (Figure 4, Figure 5 and Figure 6).

### 3.4. Effect of S. mangifera Ethanolic Extract on the Mount, Intermission, and Ejaculatory Latency

**Figure 7:**
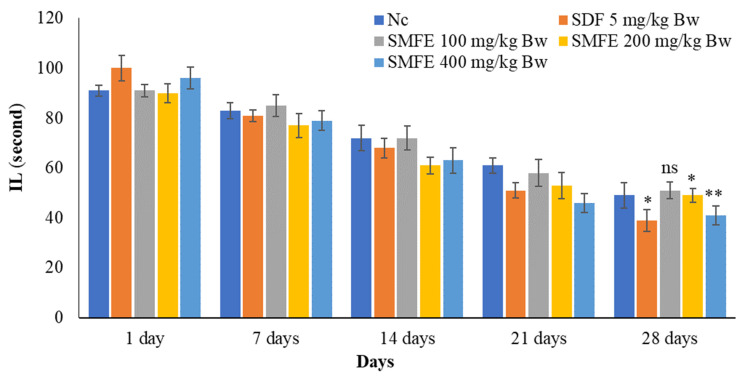
Effect of *S. mangifera* fruit extract on intromission latency (*n* = 6). The values are means ± SEM; * *p* < 0.05, ** *p* < 0.1; ^ns^ *p* > 0.05 (followed by Student Dunnett test).

**Figure 8:**
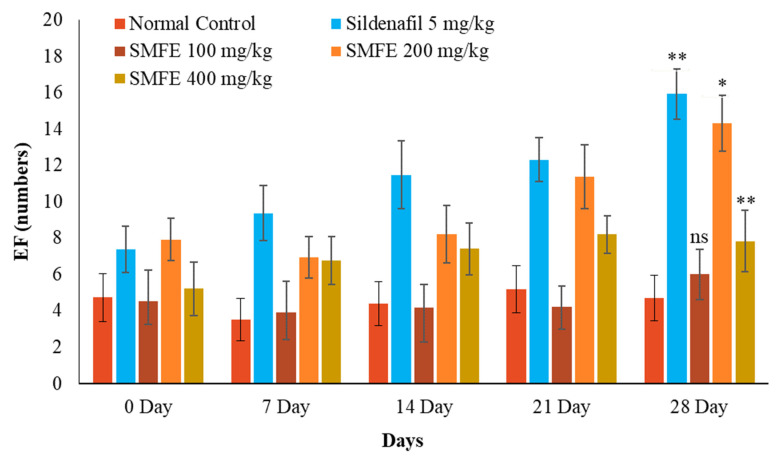
Effect of *S. mangifera* fruit extract on ejaculation frequency (*n* = 6). The values are means ± SEM; * *p* < 0.05, ** *p* < 0.1; ^ns^ *p* > 0.05 (followed by Student Dunnett test).

**Figure 9:**
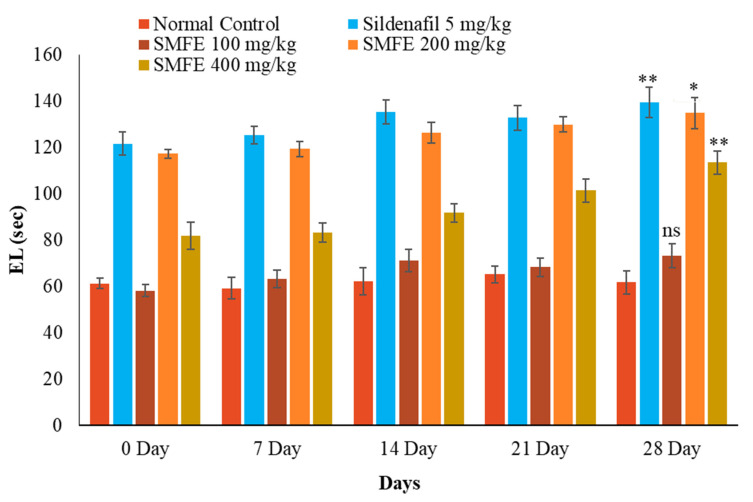
Effect of *S. mangifera* fruit extract on ejaculation latency (*n* = 6). The values are means ± SEM; * *p* < 0.05, ** *p* < 0.1; ^ns^ *p* > 0.05 (followed by Student Dunnett test).

With the administration of standard sildenafil citrate (5 mg/kg/d bodyweight), the ML and IL significantly (*p* < 0.1) decreased, and EL significantly (*p* < 0.1) increased when compared with normal control animals. Next, the ethanolic extract of *S. mangifera* fruits was administered at 400 mg/kg/d bodyweight. The ML and IL significantly (*p* < 0.01) decreased, and EL significantly (*p* < 0.01) increased; however, 200 mg/kg/d bodyweight showed a less significant (*p* < 0.05) effect on the above arousal parameters, whereas 100 mg/kg/d bodyweight did not show a significant (*p* > 0.05) effect when compared with normal control group animals (Figure 7, Figure 8 and Figure 9).

### 3.5. PDE-5 Activity

**Figure 10:**
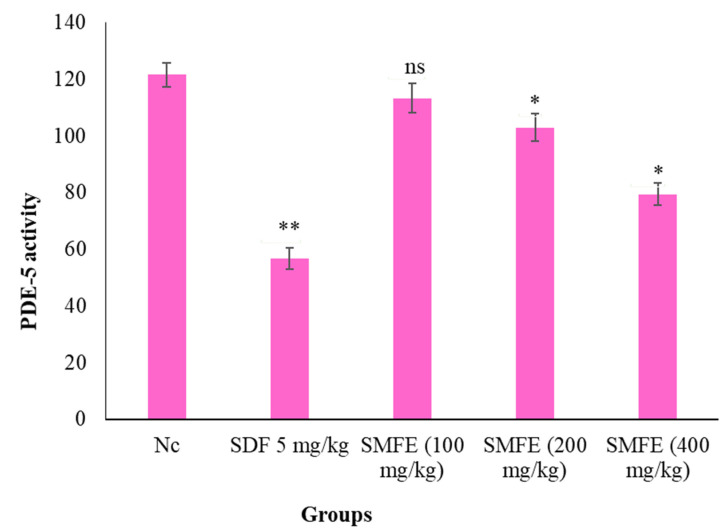
Effects of SMFE on phosphodiesterase level (*n* = 6). The values are means ± SEM; * *p* < 0.05, ** *p* < 0.1; ^ns^ *p* > 0.05.

The phosphodiesterase activity in experimental mice was revealed as dose-dependent (Figure 10). The group treated with *S. mangifera* fruit extract (SMFE) of 400 mg/kg bodyweight showed significantly decreased (*p* < 0.1) PDE-5 activity; however, in groups treated with 200 mg/kg/d bodyweight, the PDE-5 showed a less significant (*p* < 0.05) effect. In the groups treated with 100 mg/kg/d bodyweight, the activity of PDE-5 did not show a significant effect on smooth muscle compared with the normal control treated group.

### 3.6. Testosterone Level

**Figure 11:**
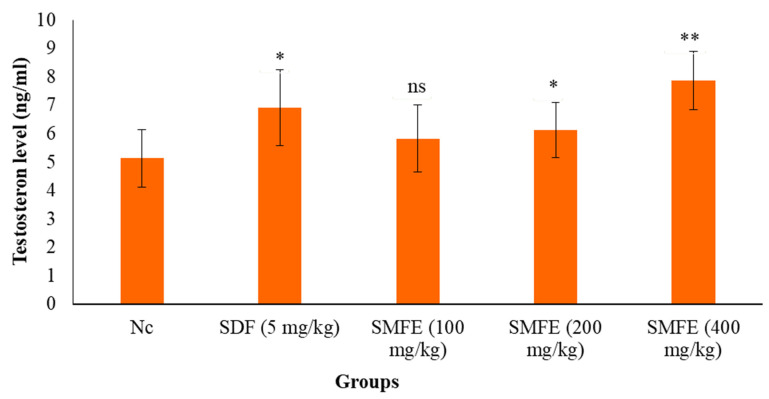
Effect of *S. mangifera* fruit extract on testosterone level (*n* = 6). The values are means ± SEM; * *p* < 0.05, ** *p* < 0.1; ^ns^ *p* > 0.05 (followed by Student Dunnett test).

The testosterone level significantly increased in sildenafil-treated groups compared with normal control groups (Figure 11). When administered 400 mg/kg/d b.w. of SMFE, the testosterone level was significantly (*p* < 0.1) increased; however, 200 mg/kg/d bodyweight of SMFE showed a less significant (*p* < 0.05) testosterone level, whereas with the administration of 100 mg/kg/d bodyweight of SMFE, the level of testosterone did not show a significant (*p* > 0.5) effect when compared with the normal group animals.

### 3.7. Nitric Oxide Level

**Figure 12:**
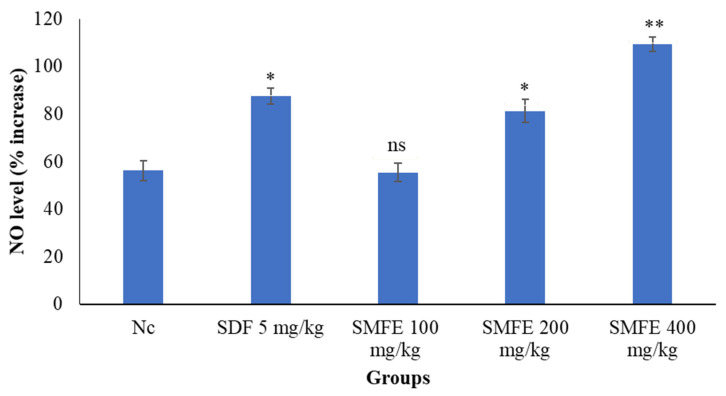
Effect of *S. mangifera* fruit extract on NO level (*n* = 6). The values are means ± SEM; * *p* < 0.05, ** *p* < 0.1; ^ns^ *p* > 0.05 (followed by Student Dunnett test).

The treated group with sildenafil significantly increased nitric oxide levels (*p* < 0.05) in comparison to normal groups (Figure 12). The nitric oxide level significantly (*p* < 0.1) increased at 400 mg/kg/d b.w. of SMFE, but at 200 mg/kg/d bodyweight of SMFE, its level was less significant (*p* < 0.05). The 100 mg/kg/d bodyweight of SMFE did not show a significant (*p* > 0.5) effect on nitric oxide compared to the normal group.

## 4. Discussion

The AutoDock automated docking approach was utilized to provide insight into the mechanism of action of the key constituents of *S. mangifera* β-amyrin, β-sitosterol, and oleanolic acid [45]. The above constituents were docked and had an excellent binding affinity with PDE-5, while the dopamine receptor D2 showed less binding affinity. Our docking methodology was validated when the co-crystallized ligand sildenafil exhibited identical binding to the crystal structure. The similarities were remarkable between β-Amyrin, oleanolic acid, and β-sitosterol in their interactions with PDE-5 in their bound forms. Hydrophobic interactions with β-amyrin, β-sitosterol, and oleanolic acid were mediated by a group of residues called Leu A: 725, Phe A: 786, and Phe A: 820. The binding of PDE-5 was dependent on these residues.

Dopamine, serotonin, and noradrenaline have all been studied by researchers for their involvement in behavioral activities, including sexual behavior [46]. Dopamine is assumed to be involved in most reward-motivated behaviors, including sexual reward, and serotonin is expected to contribute to emotions of pleasure and well-being. In contrast, noradrenaline enhances arousal and attention and promotes vigilance [47,48].

Based on *in vivo* studies, various natural sources have been used to treat different diseases in recent years. Since natural medicine is safe and does not produce harmful effects on human health, plant sources have great importance and contributions to human health, especially infertility [8]. Several plant metabolites containing marketed products are available to treat male and female infertility [49]. Researchers have reported that various secondary plant metabolites such as alkaloid, glycoside, flavonoids, phenolic, and saponins were used to increase the aphrodisiac activities, and some of the natural bioactive compounds to boost sex hormones, which is helpful for the treatment of infertility [50]. Some natural compounds, especially saponins, serve as a precursor for testosterone, progesterone, etc., increasing the body’s sex hormones (LH and FSH) [51]. Various bioactive constituents, such as saponins, alkaloids, etc., work as non-selective phosphodiesterase inhibitors [52]. The flavonoid and phenolic constituents also have an inhibitory action on phosphodiesterase and are used to treat sexual dysfunction [45]. Therefore, the presence of various bioactive molecules and β-amyrin, β-sitosterol, and oleanolic acid in the extract of *S. mangifera* might enhance sexual arousal through central or peripheral nerve activation.

The sexual behaviors were attributed to secondary plant metabolites such as alkaloids, saponins, flavonoids, and phenolics in the plant materials. During the experimental study of up to 28 days, the behavioral parameters revealed that the ethanolic extract of *S. mangifera* fruits significantly increased the mount, intromission, and ejaculation frequency, while the mount and intromission latency were significantly decreased. Still, ejaculatory latency significantly increased in experimental animals at a 400 mg/kg bodyweight dose. In contrast, these behavioral effects were less than the sildenafil-treated groups (standard group animals). Fouche et al. also revealed that the *Monsonia angustifolia* aerial part aqueous extract enhanced the aphrodisiac activity [53]. Agmo (1997) also studied the mount and intromission frequency as valuable parameters for libido and potency [54]. Increased anterior pituitary hormones and testosterone levels, which drive dopamine receptor production, might cause increased libido [55]. Furthermore, it suggested that libido enhancement may be due to bioactive phytoconstituents in the extract since they have been reported to alter androgen levels [56].

In addition, an erection is impossible without stimulating the penile muscle, which also prevents the occurrence of intromission [12]. In the present work, *S. mangifera* fruit extract (400 mg/kg bodyweight) increased the intromission frequency (IF). It means the penile muscle was activated. Cholesterol is one of the precursors for synthesizing steroidal hormones such as testosterone and bile. Therefore, increasing the cholesterol levels in the serum or testicles enhanced the aphrodisiac activity [57]. The present study observed that the ethanolic extract of *S. mangifera* fruits increased cholesterol concentrations (Figure 11), corroborating the increased testosterone concentrations. Therefore, the sexual desire may be due to increased cholesterol, serum testosterone, and anterior pituitary hormones. These hormones significantly stimulate dopamine, causing sexual desire and coitus in males [58].

**Figure 13:**
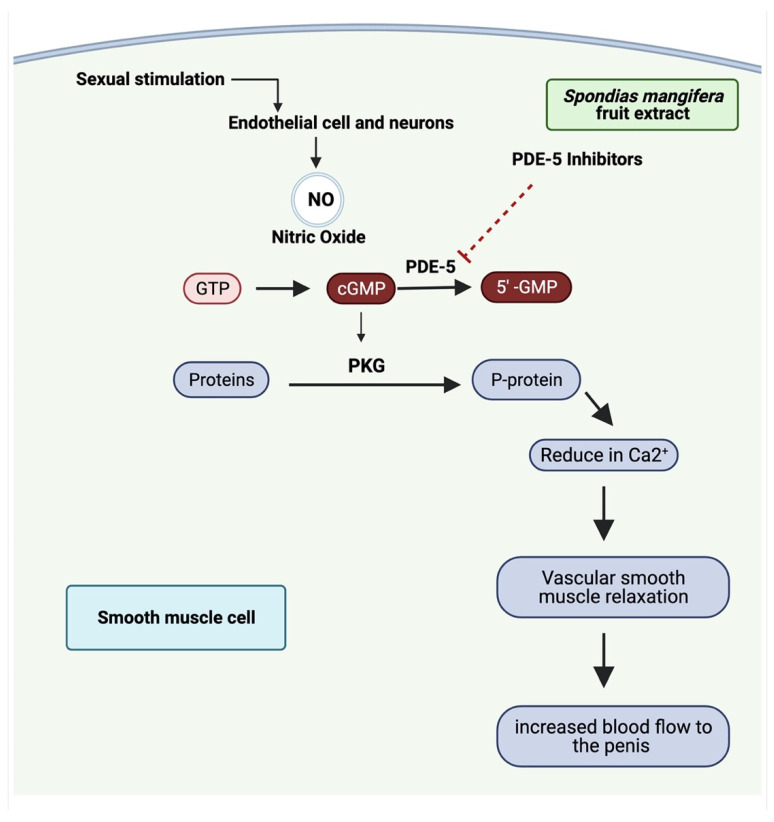
The possible mechanism of smooth muscle relaxation through PDE5 inhibition.

During the coitus, nitric oxide is secreted through the parasympathetic nerve, which activates the guanylate cyclase by entering the smooth muscle present beneath the arteries of the corpora cavernosum. The guanosine triphosphate (GTP) converted into cyclic guanosine monophosphate (cGMP) by the influence of activated sGC results in muscle relaxation and increased blood flow to the penis [32,59]. The possible mechanism of smooth muscle relaxation through PDE5 inhibition is shown in Figure 13. However, PDE-5 diminishes the levels and activity of cGMP by degradation. Various PDE-5 inhibitors are available for the treatment of erectile dysfunction. Natural medicine has shown to be a potential treatment option for male erectile dysfunction [60]. PDE-5 inhibitor (sildenafil) improved erectile function in animal groups due to altered NO/cGMP pathway [61]. Moreover, the rapid degradation of cGMP caused by the increased activity of PDE-5 impairs the proper erection since cGMP facilitates the process and relaxes smooth muscle. In the present work, ethanolic extract of *S. mangifera* fruits (400 mg/kg bodyweight) lowered the phosphodiesterase activity attributed to bioactive phytoconstituents in the extract. This study’s limitation is that it cannot determine whether or not *S. mangifera* fruits affect anxiety-induced sexual dysfunction. This necessitates further research to assess the impact of *S. mangifera* fruits on the anxiety-induced sexual dysfunction through the *in vivo* model.

## 5. Conclusions

In conclusion, the results of this research provide substantial support for the use of *S. mangifera* fruit in the treatment of erectile dysfunction. To successfully treat erectile dysfunction via PDE-5 inhibition, natural compounds must possess drug-likeness properties and a high degree of selectivity. *S. mangifera* fruit’s main constituents, β-amyrin, β-sitosterol, and oleanolic acid, had a high affinity for phosphodiesterase-5 (PDE-5), much like sildenafil as shown in our *in-silico* docking results. That has been further validated through *in-vitro* and *in-vivo* evaluation. The rise in nitric oxide and testosterone levels and the improvement in sexual behaviors (MF, IF, EF, and EL) demonstrated an excellent erotic effect. Overall, ethanolic fruit extract of *S. mangifera* has shown strong aphrodisiac potential, so that it might be employed in erectile dysfunction as an effective new drug delivery agent. However, further research is needed to determine the plant’s safety and effectiveness in sexual disorders. In addition, a thorough mechanistic investigation is necessary to explain the specific mode of action of SMEE as a libido-boosting drug candidate.

## Figures and Tables

**Table 1:** Summary of the selected constituents and the reference compound sildenafil into the active site of PDE-5, chemical structures, AutoDock vin docking scores, hydrogen bond interactions, and close contact residues.

Ligands	Chemical Structure	Binding Energy Score (kcal/mol)	H-Bond Interaction	Hydrophobic Interaction
Sildenafil	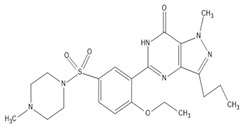	−10.01	ARG A: 667, ASN A: 662, GLN A: 817	HIS A: 613, LEU A: 725, LEU A: 765, ALN A: 765, TYR A: 612, GLN A: 725, ILE A: 768, VAL A: 782, PHE A: 820, PHE A: 786, MET A: 816, LEU A: 805, HIS A: 657, GLU A: 682, HIS A: 685, MET A: 681, VAL A: 660
Oleanolic acid	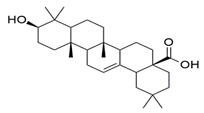	−10.51	SER A: 663	LEU A: 765, LEU A: 725, ILE A: 824, PHE A: 820, ALA A: 823, GLY A: 819, GLN A: 666, MET A: 816, ASN A: 662, ILE A: 665, LEU A: 804, PHE A: 786, ASN A: 661, HIS A: 613
β-sitosterol	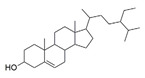	−9.90	--	LEU A: 765, LEU A: 725, VAL A: 782, ILE A: 824, LEU A: 804, PHE A: 820 ALA A: 823, GLY A: 819, MET A: 816, ILE A: 665, SER A: 663, ASN A: 662, PHE A: 786, HIS A: 613
β-amyrin	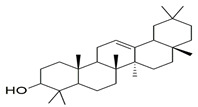	−11.61	MEH A: 816	ARG A: 667, ASN A: 661, ASN A: 662, SER A: 663, ILE A: 665, LEU A: 804, GLY A: 819, PHE A: 820, ILE A: 824, PHE A: 786, LEU A: 765, TYR A: 612, ASP A: 764, HIS A: 613, LEU A: 725

**Table 2:** Summary of the selected constituents and the reference compound sildenafil into the active site of dopamine R2, chemical structures, AutoDock vin docking scores, hydrogen bond interactions, and close contact residues.

Ligands	Chemical Structure	Binding Energy Score (kcal/mol)	H-Bond Interaction	Hydrophobic Interaction
Sildenafil	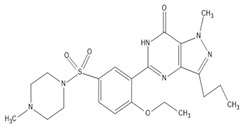	−9.2	ASP A: 114 HIS A: 393	PRO A: 405, SERA: 409, TRPA: 413, PHEA: 110, LEUA: 94, TRPA: 100, THRA: 412, VAL A: 91, TYR A: 416, VAL A: 115, SER A: 193, PHE A: 389, PHE A: 189, VAL A: 190, ILE A: 184, TYR A: 408
Oleanolic acid	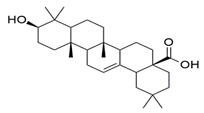	−8.6	TYR A: 213	ASP A: 1072, TYR A: 1088, ALA A: 1093, ARG A: 1096, ALA A: 1097, ILE A: 1100, TRP A: 1158, ARG A: 220, ILE A: 1003, VAL A: 1075, LYS A: 221, VAL A: 1071, ARG A: 217
β-Sitosterol	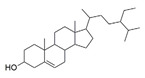	−8.9	--	VAL A: 91, TRP A: 413, GLU A: 95, LEU A: 94, LEU A: 41, SER A: 409, THR A: 412, TRP A: 100, PHE A: 489, HISA: 393, PHE A: 189, ILE A: 184, VAL A: 190, TYR A: 208, ALA A: 185, ASN A: 396, TYRA: 416,
β-Amyrin	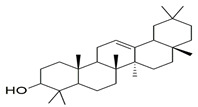	−8.6		TRP A: 413, VAL A: 91, LEU A: 94, SER A: 409, THR A: 412, TYR A: 402, ASN A: 396, PRO A: 405, TRP A: 95

## Data Availability

All data used to support the findings of this study are included within the article.
